# Optimization of Liposomal Lipid Composition for a New, Reactive Sulfur Donor, and *In Vivo* Efficacy Studies on Mice to Antagonize Cyanide Intoxication

**DOI:** 10.1155/2011/928626

**Published:** 2011-12-01

**Authors:** Ilona Petrikovics, Prashanth Jayanna, Jonathan Childress, Marianna Budai, Sarah Martin, Galina Kuzmitcheva, Gary Rockwood

**Affiliations:** ^1^Department of Chemistry, Sam Houston State University, Huntsville, TX 77341, USA; ^2^United States Army Medical Research Institute of Chemical Defense, Aberdeen Proving Ground, MD 21010, USA; ^3^Triesta Sciences, HealthCare Global Enterprises Ltd, P. Kalinga Rao Road, Sampangiramnagar, Bengaluru 560 027, India; ^4^Department of Pharmaceutics, Semmelweis University, Hőgyes Endre Street 7, H-1092 Budapest, Hungary

## Abstract

Present studies have focused on a novel cyanide antidotal system, on the coencapsulation of a new sulfur donor DTO with rhodanese within sterically stabilized liposomes. The optimal lipid composition for coencapsulation of DTO with rhodanese is the combination of 1-palmitoyl-2-oleoyl-sn-glycero-3-phosphocholine, cholesterol, cationic lipid (DOTAP), and 1,2-dipalmitoyl-sn-glycero-3-phosphoethanolamine-N-[methoxy(polyethylene glycol)-2000] ammonium salt (with molar ratios of 82.7 : 9.2 : 3.0 : 5.1). With the optimized compositions, prophylactic and therapeutic *in vivo* efficacy studies were carried out in a mice model. When DTO was coencapsulated with rhodanese and thiosulfate the prophylactic antidotal protection was 4.9 × LD_50_. Maximum antidotal protection against cyanide intoxication (15 × LD_50_) was achieved with coencapsulated rhodanese and DTO/thiosulfate in combination with sodium nitrite. When applied therapeutically, 100% survival rate (6/6) was achieved at 20 mg/kg cyanide doses with the encapsulated DTO-rhodanese-thiosulfate antidotal systems with and without sodium nitrite. These data are indicating that the appropriately formulated DTO is a promising sulfur donor for cyanide antagonism.

## 1. Introduction

The specific treatment of cyanide (CN) intoxication means the use of scavengers (e.g., methemoglobin former sodium nitrite (SN) or cobalt compounds or cyanohydrin formers, hydroxocabalamin (Cyanokit has been approved in the US), cobinamide [1], and/or the conversion of CN to the less toxic thiocyanate (SCN) with exogenously administered sulfane sulfur and sulfurtransferase enzymes [2–4]. Rhodanese (Rh) is the best characterized multifunctional, mitochondrial sulfurtransferase [5–8] catalyzing the transfer of a sulfane sulfur atom from a donor molecule to cyanide. Determining the exact role of nitrite in cyanide antagonism is not clearly understood yet. Earlier studies were focusing on the methemoglobin-forming effect of nitrite that act as a scavenger by forming a relative stable complex of cyanomethemoglobin [3, 4]. Very recent studies are focusing on the mitochondria-linked mechanism of nitrite as a nitric oxide donor [9–11].

Extensive researches are also focusing on developing effective sulfur-containing compounds serving as sulfur donors for reacting with CN with or without Rh. Thiosulfate (TS) is the classical sulfur compound found to participate in the enzyme reaction [3, 4, 12]. However, TS has limited ability to reach the endogenous Rh enzyme because of a nearly exclusive extracellular distribution [13]. Baskin et al., reported results on the efficacy of various sulfur donors demonstrating that altering the chemical substituent of the longer chain sulfide modified the ability of the candidate molecule to protect against CN toxicity [14].

 Earlier investigations were focused on administration of free Rh and the sulfur donor (SD) directly into the bloodstream [15–18]. Unfortunately, the free Rh enzyme was rapidly destroyed by the body's immune system, which makes the efficacy of this approach quite limited. To overcome the limitations for the circulating free Rh, micro- or nanosized carrier systems among others sterically stabilized unilamellar liposomes of ~100–150 nm diameter are in the focus of recent encapsulation efforts [19]. The encapsulation of Rh with a sulfur compound into liposomes—the so-called coencapsulation—can offer further advantages. Over stability enhancement for Rh the coencapsulation can provide better overall conversion of CN, since the basis component for enzyme reaction, the sulfur donor no longer has to penetrate the liposome membrane.

The lipid composition has a significant impact on the encapsulation efficiency of the Rh and/or sulfur compound and on the *in vivo* stability and antidotal effect of the carrier system [19]. Thus, optimization of the liposomal composition is an inevitable step in the design of novel antidotal systems.

Present work deals with a new lipophilic sulfur-containing compound, developed at the US Army Medical Research Institute of Chemical Defense, called DTO. In order to achieve the highest CN antidotal protection, the liposomal encapsulation of DTO with and without Rh was examined.

The objectives of this study are (1) optimization of the liposomal encapsulation for the new sulfur donor, DTO, with superior sulfur donor reactivity to the present therapy TS; (2) *in vivo *efficacy study of the coencapsulated DTO with Rh in combination with sodium nitrite on mice.

## 2. Materials and Methods

All chemicals employed were of the highest purity commercially available: potassium cyanide, TS, sodium nitrite, phosphate buffer components, ethanol, sodium chloride, concentrated hydrochloric acid, and sodium hydroxide were purchased from J. T. Baker, (Phillipsburg, NJ), formaldehyde and ferric nitrate were purchased from Fisher Scientific (Pittsburgh, PA). The liposome components (1-palmitoyl-2-oleoyl-sn-glycero-3-phosphocholine (POPC), 1, 2-dioleoyl-sn-glycero-3-phosphocholine (DOPC), N-[1-(2,3-dioleoyloxy)propyl]-N,N,N-trimethylammonium chloride (DOTAP), soy lecithin (LEC), cholesterol (Chol)) and Rh were purchased from Sigma-Aldrich (St. Louis, MO), and 1,2-dipalmitoyl-sn-glycero-3-phosphoethanolamine-N-[methoxy(polyethylene glycol)-2000] ammonium salt (PEG-PE-2000) was purchased from Avanti Polar Lipids (Alabaster, AL). Bio-Rad Protein Kit was purchased from Bio-Rad Life Sciences Laboratories, Hercules, CA.

### 2.1. Animals

Male (CD-1) mice (Charles River Breeding Laboratories, Inc., Wilmington, MA) weighing 18–20 g were housed at 21°C and in light-controlled rooms (12 h light/dark, full-spectrum lighting cycle with no twilight), and were furnished with water and 4% Rodent Chow (Teklad HSD, Inc., CITY, WI)* ad libitum*. All animal procedures were conducted in accordance with the guidelines by The Guide for the Care and Use of Laboratory Animals (National Academic Press, 1996), accredited by AAALAC (American Association for the Assessment and Accreditation of Laboratory Animal Care). At the termination of the experiments, surviving animals were euthanized in accordance with the 1986 report of the AVMA Panel of Euthanasia [20].

### 2.2. Preparation of Sterically Stabilized Liposomes (SL) Encapsulating DTO (SL-DTO), or Rh (SL-Rh), or Both of Them (SL-DTO-Rh) with and without Coencapsulated TS

POPC, DOPC, DOTAP, LEC, PEG-PE-2000, and Chol dissolved in ethanol were applied in various molar ratios in order to determine the optimal lipid composition.

The liposomes were prepared by the thin-film hydration method [21]. DTO was codissolved with the lipids. As hydrating solution isotonic phosphate buffer (pH = 7.4; osmolarity = 290 mosm) was added to the dry lipid films. Four different Rh concentrations (0.25 mg/mL, 0.50 mg/mL, 1.00 mg/mL and 1.67 mg/mL), and four different DTO concentrations (2.0; 10.0; 20.0 and 30.0 mM) were investigated with various liposomal lipid compositions to evaluate the effects of these parameters on encapsulation efficiency for Rh and/or DTO. The total lipid concentration (lipids and Chol together) was 10.0 mg/mL. In order to obtain a homogenous population of small unilamellar vesicles (SUV), the multilamellar vesicles (MLV) were extruded through polycarbonate filters (100 and 400 nm) with an extruder (Avanti Polar Lipids Inc. Alabaster, AL). Extrusions were repeated five times for each membrane unless otherwise indicated.

### 2.3. Determination of Rhodanese Activity

The formation of SCN from CN was measured spectrophotometrically (Genesys 10UV, Thermo Electron Corporation, Waltham, MA) by the method of Westley [22], with minor modifications of Petrikovics et al. [23]. One unit of Rh was defined as the amount of enzyme that forms 1 *μ*mol of SCN in 1 min.

### 2.4. Sulfur Donor Reactivity

Formation of SCN from CN with the investigated sulfur donors of TS and DTO were determined spectrophotometrically by the method of Westley [22] with minor modifications of Petrikovics et al. [23].

### 2.5. Optimal Rh Load for SL-Rh

Four different Rh concentrations (0.25 mg/mL, 0.50 mg/mL, 1.00 mg/mL, 1.67 mg/mL) were employed with a lipid composition of POPC : Chol : PEG-PE-2000 with and without DOTAP. Percentage of Rh incorporation within the liposomes was determined by the Bradford Assay [24].

### 2.6. Optimal Lipid Composition for Liposomal Rh Encapsulation

Optimal lipid composition for Rh encapsulation was determined based on the highest enzyme activity achieved by the same encapsulation process with various lipid compositions. Unencapsulated Rh was separated from SL-Rh by gel filtration on a G-100 Sephadex gel column (0.7 cm × 10 cm; GE Healthcare BioSciences AB, Sweden). Measurements were carried out in isotonic phosphate buffer at pH = 7.4. Rh activity for the fractions was determined as described above.

(1) $$\begin{array}{cc} & \text{Encapsulation}\;\text{efficiency}\;\left(\%\right)\\ & \;\;=\;\frac{\text{activity}\;\text{of}\;\text{SL-Rh}}{\text{total}\;\text{Rh}\;\text{activity}}\;\times \;100. \end{array}\;$$

For the spectrophotometric assays, 50 *μ*L liposomal samples were used. All measurements were performed at least in triplicate.

### 2.7. Optimal Lipid Composition Determination for SL-DTO

The encapsulation efficiency for the sulfur donor DTO was determined by the Rh assay described above with constant Rh concentration. When Rh concentration was constant, the rate of formation of SCN was directly proportional to the sulfur donor concentration.

(2) $$\begin{array}{cc} & \text{Encapsulation}\;\text{efficiency}\;\left(\%\right)\\ & \;\;=\frac{\text{concentration}\;\text{of}\;\text{encapsulated}\;\text{DTO}}{\text{total}\;\text{DTO}\;\text{concentration}}\;\times 100. \end{array}$$

### 2.8. Optimal Lipid Composition Determination for SL-Rh-DTO

Formation of SCN by SL-Rh-DTO with various lipid compositions was measured spectrophotometrically as described above.

(3) $$\begin{array}{cc} & \text{Encapsulation}\;\text{Efficiency}\;\left(\%\right)\\ & \;\;=\;\frac{\text{SCN}\;\text{formation}\;\text{by}\;\text{the}\;\text{given}\;\text{SL-Rh-DTO}}{\text{SCN}\;\text{formation}\;\text{by}\;\text{the}\;\text{original}\;\left(\text{before}\;\text{encapsulation}\right)\;\text{Rh}\;\text{and}\;\text{DTO}\;\text{concentration}}\;\times 100. \end{array}$$

### 2.9. Prophylaxis against CN in Mice Using SL-Rh, SL-DTO, SL-DTO-TS, SL-DTO-Rh, and SL-DTO-TS-Rh in Combination with SN

Experimental animals received KCN after pretreatment with antagonist(s) (sulfur donors and/or Rh and/or SN). Freshly prepared SL-DTO, SL-DTO-TS, SL-DTO-Rh, and SL-DTO-TS-Rh were administered intravenously (iv) by tail vein injection to mice 10 min prior to receiving CN (sc). Using 10 mL/kg doses of (SL-DTO-Rh; Table 4 experiment 5) or (SL-DTO-TS-Rh; Table 4 experiments 6 and 7) the animals received 15–20 units of Rh. Employing 10 mL/kg injections of (SL-DTO; Table 4 experiment 2) or (SL-DTO-TS; Table 4 experiments 3 and 4) the dose for DTO was 11.5 mg/kg and 14.2 mg/kg for the coencapsulated TS. SN (100 mg/kg; sc), was injected 45 min prior to CN (sc) injection (Table 4 experiments 4 and 7). The animals were evaluated 24 hours after CN exposure for mortality; surviving animals were observed for an additional week for late-developing toxicity. No toxic effects which could be attributed to SL-DTO, SL-DTO-TS, SL-DTO-Rh, SL-DTO-TS-Rh, TS, or SN (when administered alone or in various combinations) were noted in any of the mice at the doses applied. LD_50_ values were determined by the Dixon up and down method [25], using 8–18 mice for each LD_50_ determination. The LD_50_ values were given for three or more experiments. The “antidotal potency ratio” (APR) is expressed as a ratio of LD_50 (mean)_ of CN with antagonists and LD_50 (mean) _ of CN without antagonists.

### 2.10. Therapeutic Protection against CN in Mice Using SL-DTO-TS and SL-DTO-TS-Rh in Combination with SN

Animals received antidotes administered intravenously one min after CN injection (sc). Doses of antidotes were the same as described above for the prophylactic experiments. The animals were evaluated 24 hours after CN exposure for mortality. Results are given as % survival (animals alive/animals total). Total numbers of animals were 6 for each therapeutic experiment for each antidotal system.

## 3. Results and Discussion 

These studies focused on the encapsulation optimization for new sulfur donor DTO when encapsulated with Rh and/or TS within sterically stabilized liposomes. The *in vitro* sulfur donor reactivity comparison shows that DTO reacts 15-times faster with CN at constant Rh concentration than TS (Table 1). Encapsulation efficiencies for both Rh and DTO were optimized as a function of Rh-load, DTO-load, and lipid composition. 

When encapsulating Rh alone, small amount of the cationic lipid DOTAP proved to be beneficial to enhance encapsulation efficiency (Table 2). The optimum Rh concentration within the liposomes was 0.25 mg/mL (Table 2). 

For the encapsulation of DTO with a concentration of 2 mM, six different liposomal compositions were examined to rule out the role of lipid composition (Table 3). Each contained PEG-PE-2000 in 5.1 mol%, lipid-to-Chol ratio was 9 to 1. Also, the cationic lipid, DOTAP in 3.0 mol%, was utilized to influence the charge of the liposomal surface in hopes that a positively charged surface would provide increased affinity for DTO. On the basis of the results it can be concluded that the presence of DOTAP leads to significant (*P* < 0.05) higher encapsulation efficiency with DOPC and POPC as main lipid component. LEC liposomes demonstrated the least encapsulation efficiency for DTO, and DOTAP appeared to provide little to no enhancement (Table 3). The role of DOTAP in enhancing the encapsulation efficiency can be explained with electrostatic interactions between DOTAP and DTO at the pH value examined (pH = 7.4). Early experiments with the 60 : 40 ratio of dipalmitoyl-phosphatidylcholine (DPPC) to Chol composition provided very low encapsulation efficiency for encapsulation of DTO (data not shown here). As DTO is a lipophilic sulfur donor, it can be expected that it is localized in the liposomal bilayer, more or less immersed in it. The saturated bonds of DPPC providing an ordered, relative rigid structure may inhibit the immersion of DTO in the liposomal membrane. Contrarily, POPC and DOPC possessing double bonds and as a consequent of it a less ordered and more fluid membrane structure can promote the encapsulation of DTO. Thus, instead of DPPC, DOPC, or POPC were used for liposome preparation and ratios were adjusted to 90 : 10 lipid to Chol. The results shown in Table 3 indicate that DOPC liposomes containing 3 mol% DOTAP provided the highest encapsulation efficiency at 81.7 ± 3.1%. POPC liposomes with 3% DOTAP were close behind with an encapsulation efficiency of 78.4 ± 2.3%. However, there was no significant difference between encapsulation efficiencies with DOPC and POPC. 

The liposome compositions including DOTAP were used in further experiments due to the increase in encapsulation efficiency achieved by these films. The effect on encapsulation efficiency by the increase in DTO concentration was evaluated for DOPC and POPC containing liposome compositions with both sets of liposomes containing 3% DOTAP. In order to evaluate the role of DTO concentration on the encapsulation efficiency each set's films were prepared with DTO concentrations of 10 mM, 20 mM, and 30 mM. 

The encapsulation efficiency remained high for each liposome formulation containing 3% DOTAP for each applied DTO concentrations of 10 mM, 20 mM, and 30 mM. The encapsulation efficiencies of DTO for POPC samples were 69.7 ± 2.3%, 82.8 ± 7.1%, 79.2 ± 8.1%, while for the DOPC samples DTO encapsulation efficiencies of 74.2 ± 2.0%, 86.2 ± 3.9%, and 89.9 ± 4.2% were determined, for 10 mM, 20 mM, and 30 mM DTO concentrations, respectively. For a given DTO concentration there was no significant difference between the encapsulation efficiency values for DOPC or POPC liposomes (*P* > 0.05). 

### 3.1. Reevaluation of Rhodanese Encapsulation

The optimal liposome composition for the encapsulation of Rh was determined in earlier experiments to be the 60 : 40 ratio of POPC to Chol [19]. However, the prior experiments did not evaluate the DOPC or the cationic lipid DOTAP. Furthermore, Rh was added either in isotonic HEPES buffer (pH = 7.4–7.7) or in 5% (w/w) aqueous solution of glucose (GLU; pH = 4.2–7.8) to the dry lipid films. For the purpose of coencapsulating DTO with Rh, the Rh encapsulation efficiency must be determined for the same lipid compositions and in the same hydrating systems as in the case of DTO. The optimal liposome composition for Rh encapsulation was the 90 : 10 ratio of POPC to Chol with the use of DOTAP. Also, the 3.0 mol% DOTAP again increased the encapsulation efficiency for most of the different liposomal compositions (Table 2). 

### 3.2. Coencapsulation of DTO and Rhodanese

For the coencapsulation of DTO and Rh, the combination of POPC, Chol, PEG-PE-2000, and DOTAP (with molar ratios of 82.7 : 9.2 : 5.1 : 3.0) was chosen as the most adequate liposome composition. The mentioned composition of sterically stabilized, positively charged liposomes performed the best in the coencapsulation, with a coencapsulation efficiency for Rh and DTO of 88.6 ± 17.1% (with a Rh load of 0.25 mg/mL and a DTO concentration of 30 mM). As the coencapsulation efficiency was determined on the basis of SCN formation by SL-Rh-DTO; the given value represents the combined effect of Rh and DTO in CN conversion. For the sake of comparison, encapsulation efficiency for the coencapsulated Rh alone—with 0.25 mg/mL concentration—was 74%, while for DTO alone—with 10 mM DTO load—was 57.7 ± 8.1%. Increasing the concentration of DTO produced similar encapsulation efficiencies, than in case of 10 mM. With DTO loads of 20 mM and 30 mM for the coencapsulated DTO encapsulation efficiencies of 55.6 ± 4.0% and 61.6 ± 17.6% were measured, respectively. The conversion of CN to SCN by the coencapsulation of 10 mM, 20 mM, and 30 mM DTO with Rh also proved to remain linear with an *R* ^2^ value of 0.9930. The ability to co-encapsulate DTO, or any other sulfur donor molecule with Rh should provide better overall conversion of CN, since the sulfur donor no longer has to penetrate the liposome membrane.

### 3.3. *In Vivo* Efficacy Testing

*In vivo* evaluation of the optimized liposomal preparations made from DTO/(DTO + TS) and/or Rh were tested as cyanide antidotes on a mice model. Based on the above optimization efforts, for further *in vivo* evaluations we employed 3% DOTAP; 0.25 mg/mL Rh load, 30 mM DTO load with the lipid composition of POPC : Chol : PEG-PE-2000 : DOTAP = 82.7 : 9.2 : 5.1 : 3.0. The *in vivo* efficacy was expressed as antidotal potency ratio (APR). 

The *in vivo* prophylactic treatment results with Rh and DTO/TS encapsulated within the optimized liposomal formulations are shown in Table 4. 

SL-DTO alone provided a protection with an APR of 2.2. (Table 4 experiment 2). This protection was enhanced (APR = 4.8) when TS was coencapsulated with DTO in a molar ratio of 1 : 1 (Table 4 experiment 3). Coencapsulation of TS with DTO is also believed to provide protection against product inhibition by sulphite (Zottolla, personal communication). As it was expected, SN further enhanced the protection with the APR of 6.7. (Table 4 experiment 4). When DTO was coencapsulated with Rh (Table 4 experiment 5), the APR was 3.9. When DTO was coencapsulated with TS and Rh, (SL-DTO-Rh) the APR was enhanced to 4.9 (Table 4 experiment 6). The highest protection (APR = 15.3) was achieved with the combination of (SL-DTO-TS-Rh) and SN (Table 4 experiment 7). Expressing the relative antidotal potency ratios (RAPR) better indicates the differences in protection with two antidotal systems (Table 5). Comparing experiment 2 and 3 (RAPR = 2.2) represents the effects of TS coencapsulation with DTO. When comparing experiments 3 and 4 (RAPR = 1.4) or experiments 6 and 7 (RAPR = 3.1) the enhancement effects reached by SN are represented. The significant enhancement by Rh is expressed when comparing experiments 2 and 5 (RAPR = 1.8) and experiments 4 and 7 (RAPR = 2.3) confirming earlier studies with other types of encapsulation formulations for Rh [23, 26, 27]. 

Table 6 shows the therapeutic antidotal protection with the (SL-DTO-TS-Rh) combinations with and without SN. At approximately 2 LD_50_ dose of KCN (15 mg/kg), all the 6 animals survived in each experiment (Table 6 experiments 1, 2, and 3). However, when the KCN dose was enhanced (20 mg/kg, approximately 3 LD_50_) the survival rate with (SL-DTO-TS) + SN was 67% (Table 6 experiment 4), while with (SL-DTO-TS) without SN provided a 50% survival rate (Table 6 experiment 6). However the (SL-DTO-TS-Rh) antidotal system with and without SN also provided a 100% therapeutic protection (Table 6 experiments 5 and 7).

## 4. Conclusions 

The present experiments and results are confirming that the approach of utilizing externally administered, encapsulated, metabolizing rhodanese may have broad implications in cyanide antidotal therapy. The application of this approach has been successfully tested in animal models. In summary, these studies are describing the prophylactic and therapeutic *in vivo* efficacy of the encapsulated Rh and the new, reactive sulfur donor DTO. Optimization efforts were attempted for the liposomal lipid compositions, Rh-load, and coencapsulation of two sulfur donors (TS and DTO) and Rh to enhance the encapsulation efficiency for the given components. Optimization of the carrier systems is always a major part of these types of research efforts. Considering the high lipophilicity of DTO, for further *in vivo* applications other introduction routes (e.g., intramuscular) with further formulation optimizations are recommended.

## Figures and Tables

**Table 1:** Comparison of *in vitro* sulfur donor reactivity of TS and DTO determined with free Rh.

Rate of CN conversion (mmol SCN/min)		Ratio
TS	DTO	DTO/TS

0.2	3.0	15.0

**Table 2:** Rh-load optimization with and without DOTAP.

Composition of liposomes	Original Rh concentration (mg/mL)	% of Rh incorporation determined by Bradford
POPC : Chol : PEG-PE-2000 = 56.9 : 38: 5.1	1.00	16.6
POPC : Chol : PEG-PE-2000 = 56.9 : 38: 5.1	0.25	26.9
POPC : Chol : PEG-PE-2000 : DOTAP = 82.7 : 9.2 : 5.1 : 3.0	1.67	18.2
POPC : Chol : PEG-PE-2000 : DOTAP = 82.7 : 9.2 : 5.1 : 3.0	0.50	55.8
POPC : Chol : PEG-PE-2000 : DOTAP = 82.7 : 9.2 : 5.1 : 3.0	0.25	74.0
POPC : Chol : PEG-PE-2000 : DOTAP = 82.7 : 9.2 : 5.1 : 3.0	1.00	52.9

**Table 3:** Encapsulation efficiencies for DTO in various liposome compositions with and without DOTAP. DTO concentration was 2.0 mM. Total lipid concentration was 10.0 mg/mL.

Liposomal composition (mol%)				Encapsulation efficiency (%) with various lipid components		
Lipid	Chol	PEG-PE-2000	DOTAP	POPC	DOPC	LEC
82.71	9.19	5.1	3.0	78.4 ± 2.3	81.7 ± 3.1	64.3 ± 3.0
85.41	9.49	5.1	—	60.7 ± 3.0	63.8 ± 1.2	61.6 ± 1.5

**Table 4:** Prophylactic protection by various cyanide antidotal combinations. APR denotes antidotal potency ratio, which can be calculated as the ratio of the average LD_50_ of CN with and without antagonists.

Exp no.	Treatment	LD_50_ (mg/kg; mean; range)	APR
1	Control	7.8 (4.6–13.1)	1
2	(SL-DTO) (iv) + CN (sc)	17.3 (9.8–30.7)	2.2
3	(SL-DTO-TS) (iv) + CN (sc)	38.0 (21.5–67.3)	4.8
4	(SL-DTO-TS) (iv) + SN (ip) + CN (sc)	52.7 (29.7–93.2)	6.7
5	(SL-DTO-Rh) (iv) + CN (sc)	30.7 (14.6–64.0)	3.9
6	(SL-DTO-TS-Rh) (iv) + CN (sc)	38.0 (22.6–64.2)	4.9
7	(SL-DTO-TS-Rh) (iv)+ SN (ip) + CN (sc)	120.0 (68.2–213.0)	15.3

**Table 5:** Relative prophylactic antidotal potency ratios (RAPRs) to express enhancing effects by Rh, SN, and coencapsulated TS.

Enhancing effects by Rh	RAPR
Comparison of exps 5 and 2	3.9/2.2 = **1.8**
Comparison of exps 7 and 4	15.3/6.7 = **2.3**

Enhancing effects by SN	RAPR
Comparison of exps 4 and 3	6.7/4.8 = **1.4**
Comparison of exps 7 and 6	15.3/4.9 = **3.1**

Enhancement by coencapsulated TS	RAPR
Comparison of exps 3 and 2	4.8/2.2 = **2.1**
Comparison of exps 6 and 5	4.9/3.9 = **1.3**

**Table 6:** Therapeutic protection by various CN antidotal combinations. Control: KCN LD_50_ = 7.8 (4.6–13.1) mg/kg.

Exp no.	KCN dose (mg/kg)	Treatment	Survival (%)
1	15	(SL-DTO-TS-Rh)	6/6 (100%)
2	15	(SL-DTO-TS) + SN	6/6 (100%)
3	15	(SL-DTO-TS-Rh) + SN	6/6 (100%)
4	20	(SL-DTO-TS) + SN	4/6 (67%)
5	20	(SL-DTO-TS-Rh)	6/6 (100%)
6	20	(SL-DTO-TS)	3/6 (50%)
7	20	(SL-DTO-TS-Rh) + SN	6/6 (100%)
